# Temporal trends and spatial analysis of leprosy surveillance indicators in the municipalities of the state of Mato Grosso, 2008-2022

**DOI:** 10.1590/0037-8682-0145-2024

**Published:** 2024-10-28

**Authors:** Lúcia Rolim Santana de Freitas, Elisabeth Carmen Duarte

**Affiliations:** 1 Universidade de Brasília, Faculdade de Medicina, Brasília, DF, Brasil.

**Keywords:** Leprosy, Ecological studies, Public health surveillance, Disease hotspot, Spatial analysis

## Abstract

**Background:**

In 2022, Mato Grosso (MT, Brazil) reported the highest detection rate of new leprosy cases (66.20 per 100,000 inhabitants) among all Brazilian states. Monitoring of leprosy indicators is an important control strategy in hyperendemic areas. We aimed to describe the temporal trends and identify clusters of municipalities according to leprosy surveillance indicators in MT between 2008 and 2022.

**Methods:**

Data from the Notifiable Diseases Information System were used to analyze new case detection rate of leprosy (NCDR), new case detection rate of leprosy among children aged <15 years (NCD15), and rate of new cases with grade 2 physical disability (G2DR). Spatial scan statistics with pure spatial analysis and spatial autocorrelation maps were used to analyze the spatial patterns. Joinpoint regression was used to estimate the annual percentage change (APC) in these indicators.

**Results:**

The NCDR decreased (APC: −20.2%, 95% confidence interval (CI): −38.7% to −7.4%) between 2019 and 2021. The NCD15 also decreased (APC: −19.2%, 95% CI: −36.4% to −10.3%) between 2018 and 2022. Conversely, the G2DR remained stable throughout the study (APC: 3.2%, 95% CI: −0.1% to 6.7%). Global Moran’s index (Moran’s I) confirmed the existence of spatial dependence among the municipalities for NCDR (Moran’s I=0.348), NCD15 (Moran’s I=0.269), and G2DR (Moran’s I=0.275). Clusters with high NCDR levels included 13 municipalities in the northern and eastern macroregions, while clusters with high G2DR levels included 12 municipalities in the northwestern, northern, and eastern macroregions.

**Conclusions:**

The NCDR and NCD15 decreased, but the G2DR remained stable between 2018 and 2022. The coronavirus disease 2019 (COVID-19) pandemic had a potential negative impact on leprosy case detection, highlighting the need to strengthen leprosy surveillance efforts. The identified clusters of MT municipalities can significantly assist in this task.

## INTRODUCTION

By 2022, approximately 174,000 new cases of leprosy were reported worldwide. The persistence of a substantial leprosy burden, along with its occasionally disabling and severe sequelae, poses a significant public health challenge in many countries worldwide, particularly in tropical and subtropical regions. Brazil accounts for 11.3% of the global total leprosy cases, making it the country with the second-highest absolute number of cases globally^1^.

However, scientific evidence has shown that leprosy can be eliminated as a public health problem^2^. As it is a curable disease, timely detection and treatment, along with contact tracing measures, can significantly improve patient prognosis, prevent the occurrence of sequelae, and reduce its transmission and the global disease burden. The World Health Organization (WHO) is leading global efforts to reduce the burden of leprosy and eliminate it as a public health problem by 2030 as part of the United Nations Sustainable Development Goals. The WHO’s global strategy, “Towards zero leprosy (2021-2030),” emphasizes integrated active case detection as a key pillar for controlling, interrupting transmission, and potentially eliminating the disease^3^. Several indicators, such as the detection rate of new leprosy cases (NCDR), the number of cases in children below 15 years of age (NCD15), and the number of cases with grade 2 physical disability at the time of diagnosis, are useful for monitoring the achievement of these objectives. These indicators not only reflect the vulnerability of the population and the disease burden, but also assess the effectiveness of surveillance efforts for leprosy control within specific communities^4^.

In Brazil, progress has been made in accordance with the global strategy proposed by the WHO^5^. However, the burden of leprosy and the coverage and quality of leprosy surveillance remain significantly uneven across the country, varying by region, state, or within state. For example, southern states have successfully achieved the goal of eliminating leprosy as a public health problem, while states in the Center-West (CO), North, and Northeast Regions still face this challenge^6^ ^-^ ^9^. Mato Grosso (MT) is located in the CO region, a hyperendemic area where the disease is actively transmitted. In 2022, MT had the highest detection rate of new cases of leprosy in the general population (66.20 new cases per 100,000 inhabitants) among all Brazilian states^10^. To effectively address this issue in MT, understanding the internal inequalities related to the determinants and factors associated with the transmission and maintenance of leprosy in this state is crucial. Additionally, enhancing the effectiveness of leprosy surveillance in different intrastate scenarios remain essential.

This study aimed to describe the temporal trends and identify clusters of municipalities based on recent leprosy surveillance indicators in MT between 2008 and 2022.

The results of this study can stimulate informed discussions and enhance the focus and effectiveness of strategic actions aimed at achieving the “Towards zero leprosy (2021-2030)” goal in MT. This evidence-based approach facilitates informed decision-making and fosters collaboration with national control targets.

## METHODS

This ecological study utilized routine disease surveillance data from MT municipalities as the unit of analysis^11^. Newly diagnosed and reported cases of leprosy between 2008 and 2022 were aggregated for each municipality.

The state of MT is located in the CO region. It has 3.7 million inhabitants (2022) and a geographical area of 903,208.361 km^2^. With an average population density of only 4 inhabitants per km², it is among the least densely populated states in Brazil^2^. This study included 141 municipalities.

Data on leprosy cases were obtained from the Notifiable Diseases Information System managed by the Brazilian Ministry of Health^13^. Population data were obtained from the Brazilian Institute of Geography and Statistics (IBGE)^11^ ^,^ ^12^, provided by the Department of Informatics of the Unified Health System. The populations from the 2010 and 2022 IBGE censuses and intercensal projections (2008-2021) were used to calculate the indicators for 2010 and 2022. Additionally, cartographic bases in digital and georeferenced formats were obtained from the IBGE website^12^.

The municipal leprosy indicators were analyzed following the “Guidelines for the Surveillance, Care, and Elimination of Leprosy as a Public Health Problem”^14^. These indicators included the average new case detection rate (NCDR) of leprosy per 100,000 inhabitants, the average new case detection rate of leprosy in children below 15 years old per 100,000 inhabitants (NCD15), and the average rate of new cases with grade 2 physical disability (G2DR) per million inhabitants. The degree of physical disability was determined through a neurological assessment of the patient’s eyes, hands, and feet^14^ ^,^ ^15^. This G2DR indicator reflects the presence of visible disabilities and deformities (hands and feet) and eyes (severe visual impairment, iridocyclitis, lagophthalmos, corneal opacity, and inability to count fingers at a distance of 6 m). It served as a proxy for late diagnosis in this study.

Joinpoint regression was used to calculate the annual variations in the indicators between 2008 and 2022. This technique employs segmented linear regression to identify points where the trend changes by fitting lines to the data and determining inflection points, assuming a Poisson distribution^16^. After the segments were identified, the annual percentage change (APC), average APC, and their respective 95% confidence intervals (CIs) were calculated and validated. These results were used to classify the trends as increasing (positive APC, p<0.05), decreasing (negative APC, p<0.05), or stable (p≥0.05).

The spatial scan statistics technique was used to identify significant clusters in the MT in 2008-2022. This approach uses a circular geographical window that covers the area of interest, as described by Kulldorff (1997)^17^. The level of statistical significance (likelihood ratio test) was set to 0.05. The clusters were identified through a purely spatial analysis, adjusting for the occurrence of cases based on the municipal population size to estimate the relative risk (RR)^18^. This estimation enabled comparisons across different areas, thereby eliminating the influence of population size on the analysis^19^.

To correct for instabilities in the calculation of local estimates of the municipal leprosy indicators, a smoothing technique was applied using a local empirical Bayesian estimator. The Bayesian method is particularly useful for estimating risks in small areas (municipalities), where data are prone to random error inflation. It incorporates spatial effects by calculating estimates locally, using data from neighboring municipalities, and converging toward a local rather than a global mean. The global Moran’s index was applied to verify the global spatial autocorrelation. This index ranges from −1 to +1, where 0 indicates no spatial autocorrelation, values close to +1 indicate positive spatial autocorrelation, and values close to −1 indicate negative spatial autocorrelation. The closer the index is to +1 or −1, the greater the similarity between neighboring areas.

To identify significant spatial groupings or *clusters*, the univariate local Moran’s index (or local indicator of spatial association)^20^ was calculated. This index identifies patterns of spatial dependence and risk. Q1 (high/high) indicates municipalities with a spatial association and a high frequency of cases and are surrounded by other municipalities with high frequencies. Q2 (low/low) indicates municipalities with a spatial association and a low frequency of cases and are surrounded by other municipalities with low frequencies. Q3 (high/low) and Q4 (low/high) indicate municipalities with no spatial association and display different case frequencies compared with their neighboring areas.

The analyses were carried out using Joinpoint software version 5.0.2 (available at https://surveillance.cancer.gov/joinpoint/) to analyze temporal trends; R version 4.3.0^21^ to construct thematic maps; GeoDa 1.22.0.4 (available at https://spatial.uchicago.edu/geoda) to calculate the spatial weight matrix and autocorrelation indicators; and SaTScan 10.1.3 software (available at https://www.satscan.org/) to detect spatial *clusters*.

This study only utilized publicly available secondary data, without identifying any of the participants. The procedures were carried out following the recommendations outlined in Resolution 510 of 2016 and Resolution 466 of 2012 from the National Health Council, which governs the principles of ethics in research involving humans in Brazil.

## RESULTS

From 2008 to 2022, 45,972 new cases of leprosy were reported in the State of MT, with an average of 3,064.8 cases per year. Overall, 5.3% (n = 2,449) of the cases were diagnosed in children aged <15 years, with an average of 162 cases per year. Grade 2 physical disability at the time of diagnosis was present in 5.2% (n = 2,374) of diagnosed individuals (Table 1).

**TABLE 1:** Number of leprosy cases and selected epidemiological indicators by year of diagnosis in Mato Grosso from 2008 to 2022.

Year	New cases	Cases in children aged <15 years	Cases with grade 2 physical disability	New case detection rate of leprosy per 100,000 inhabitants	New case detection rate of leprosy among children aged <15 years per 100,000 inhabitants	Rate of new cases with grade 2 physical disability per 1,000,000 inhabitants
2008	2,811	184	95	95.04	22.46	32.12
2009	2,752	152	107	91.68	18.59	35.65
2010	2,655	151	127	87.48	19.37	41.84
2011	2,790	171	132	90.70	21.63	42.91
2012	2,690	142	134	86.35	17.73	43.01
2013	2,939	182	154	92.36	22.65	48.40
2014	3,140	217	169	97.38	27.02	52.41
2015	3,119	194	141	95.51	24.09	43.18
2016	2,877	170	117	87.04	21.05	35.40
2017	3,520	195	162	105.25	24.06	48.44
2018	4,791	196	209	139.19	24.04	60.72
2019	4,607	192	307	132.22	23.45	88.11
2020	2,640	104	161	74.87	12.68	45.66
2021	2,162	95	151	60.61	11.57	42.33
2022	2,479	104	208	67.76	12.56	56.85
**Total**	**45,972**	**2,449**	**2,374**	**93.56** ^a^	**20.20** ^a^	**47.80** ^a^

**a:** Average rate.

The prevalence of NCDR decreased from 95.04 to 67.76 new cases per 100,000 inhabitants from 2008 to 2022. This reduction was significant from 2019 to 2021, with an annual percentage change (APC) of −20.2% (95% CI: −38.7% to −7.4%) in this period (Tables 1 and 2). NCD15 decreased from 22.46 to 12.56 new cases per 100,000 inhabitants from 2008 to 2022. This reduction was significant from 2018 to 2022, with an APC of −19.2% (95% CI: −36.4% to −10.3%).

Conversely, G2DR varied from 32.12 to 56.85 cases per million inhabitants from 2008 to 2022. This difference was not significant, indicating stability throughout the study period (APC: 3.2%, 95% CI: −0.1% to 6.7%) (Tables 1 and 2).

**TABLE 2:** Joinpoint regression analysis of selected leprosy epidemiological indicators in Mato Grosso from 2008 to 2022.

Indicator	Annual percentage change (APC)			Average annual percentage change (AAPC)		
	Periods	APC	CI (95%)	Full period	AAPC	CI (95%)
New case detection rate of leprosy per 100,000 inhabitants	2008-2019	2.5*	(0.2 to 6.6)	2008-2022	−2.9*	(−5.5 to −0.4)
	2019-2022	-20.2*	(−38.7 to −7.4)			
New case detection rate of leprosy among people under 15 years old per 100,000 inhabitants	2008-2018	2.2	(−0.6 to 6.7)	2008-2022	−4.4*	(−7.4 to −2.1)
	2018-2022	-19.2*	(−36.4 to −10.3)			
Rate of new cases with grade 2 physical disability per 1,000,000 inhabitants	2008-2018	3.2	(−0.1 to 6.7)	2008-2022	3.2	(−0.1 to 6.7)

*Significantly different from 0 (p<0.05). **CI (95%):** 95% confidence interval.

In this analysis, covering the period from 2008 to 2022, the spatial scan statistics identified the following clusters of significant municipalities for each of the indicators analyzed: 11 clusters for NCDR, 5 for NCD15, and 8 for G2DR (Table 3). Two clusters within the same primary municipalities were identified as overlapping across all three epidemiological indicators: the Peixoto de Azevedo and Juína clusters. For the NCD15 and G2DR indicators, two other overlapping clusters were identified within the same primary municipality: the Juína and Sinop clusters (Table 3). Notably, these clusters represent some of the most significant findings in this study.

**TABLE 3:** Statistics of the clusters of significant municipalities, according to selected epidemiological indicators of leprosy in Mato Grosso from 2008 to 2022^a^.

Indicator	Cluster-Central municipality	Number of municipalities	Relative risk	p-value
New case detection rate of leprosy per 100,000 inhabitants	1 - Peixoto de Azevedo	17	2.23	<0.001
	2 - União do Sul	12	2.52	<0.001
	3 - Juína	2	3.82	<0.001
	4 - Canarana	7	2.39	<0.001
	5 - Paranaíta	18	1.68	<0.001
	6 - Ribeirãozinho	1	4.10	<0.001
	7 - Santo Antônio de Leverger	1	1.77	<0.001
	8 - Araputanga	1	1.52	<0.001
	9 - Mirassol d'Oeste	1	1.39	<0.001
	10 - São José do Rio Claro	1	1.29	0.004
	11 - Rosário Oeste	1	1.26	0.037
New case detection rate of leprosy among people under 15 years old per 100,000 inhabitants	1 - Peixoto de Azevedo	11	2.24	<0.001
	2 - Querência	17	2.63	<0.001
	3 - Juína	2	4.20	<0.001
	4 - Alta Floresta	1	3.25	<0.001
	5 - Sinop	18	1.31	<0.001
Rate of new cases with grade 2 physical disability per 1,000,000 inhabitants	1 - Juína	2	6.65	<0.001
	2 - Peixoto de Azevedo	11	3.35	<0.001
	3 - Nova Monte Verde	8	1.80	<0.001
	4 - Tabaporã	7	1.60	<0.001
	5 - Mirassol d'Oeste	1	2.50	<0.001
	6 - Jauru	1	3.23	<0.001
	7 - Ponte Branca	1	7.42	<0.001
	8 - Sinop	4	1.39	0.034

a Clusters estimated using the spatial scan statistic.

**FIGURE 1:**
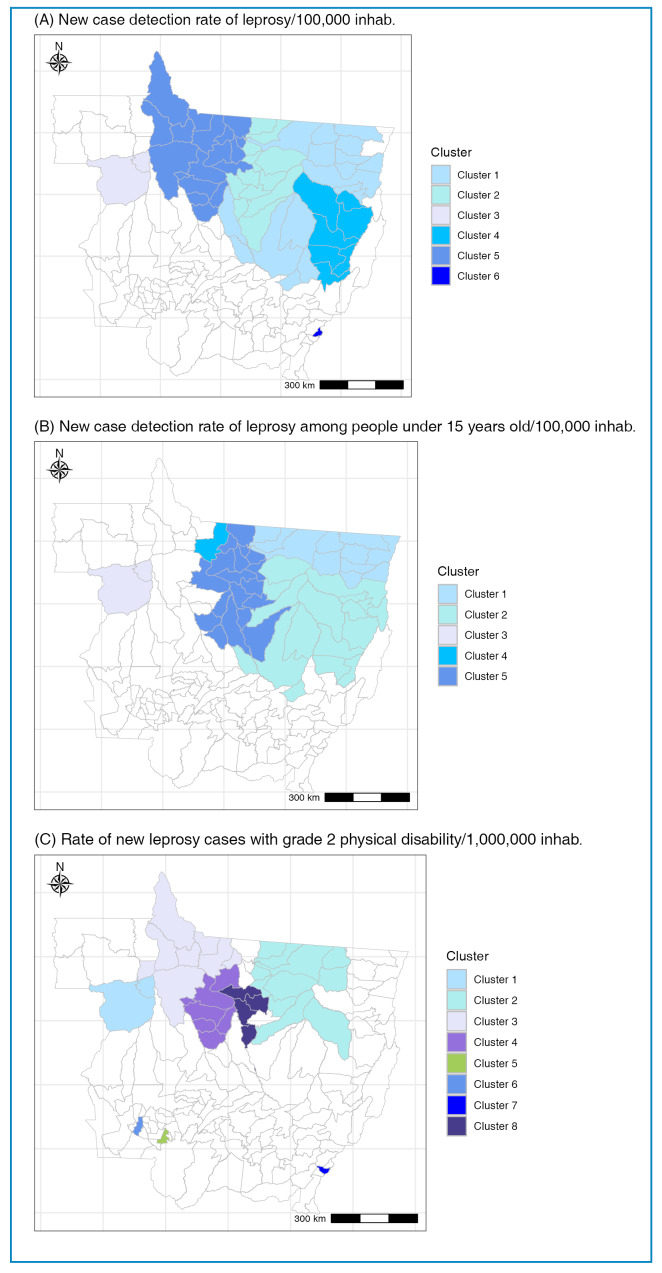
Spatial distribution of clusters of significant municipalities according to the selected epidemiological indicators of leprosy in Mato Grosso from 2008 to 2022^a^.

^a^Clusters estimated using the spatial scan statistic.

Considering the NCDR, NCD15, and G2DR indicators for the period analyzed (2008-2022), the municipalities with the highest leprosy burdens were predominantly located in the northwestern, northern, and eastern macroregions of MT (Figure 1). Clusters were also prominent in the municipalities of Ribeirãozinho and Ponte Branca, located in the eastern macroregion of the state, which demonstrated high NCDR (RR = 4.10) and G2DR (RR = 7.42) values, respectively. However, no significant clusters for NCD15 were identified in these municipalities throughout the study.

**FIGURE 2:**
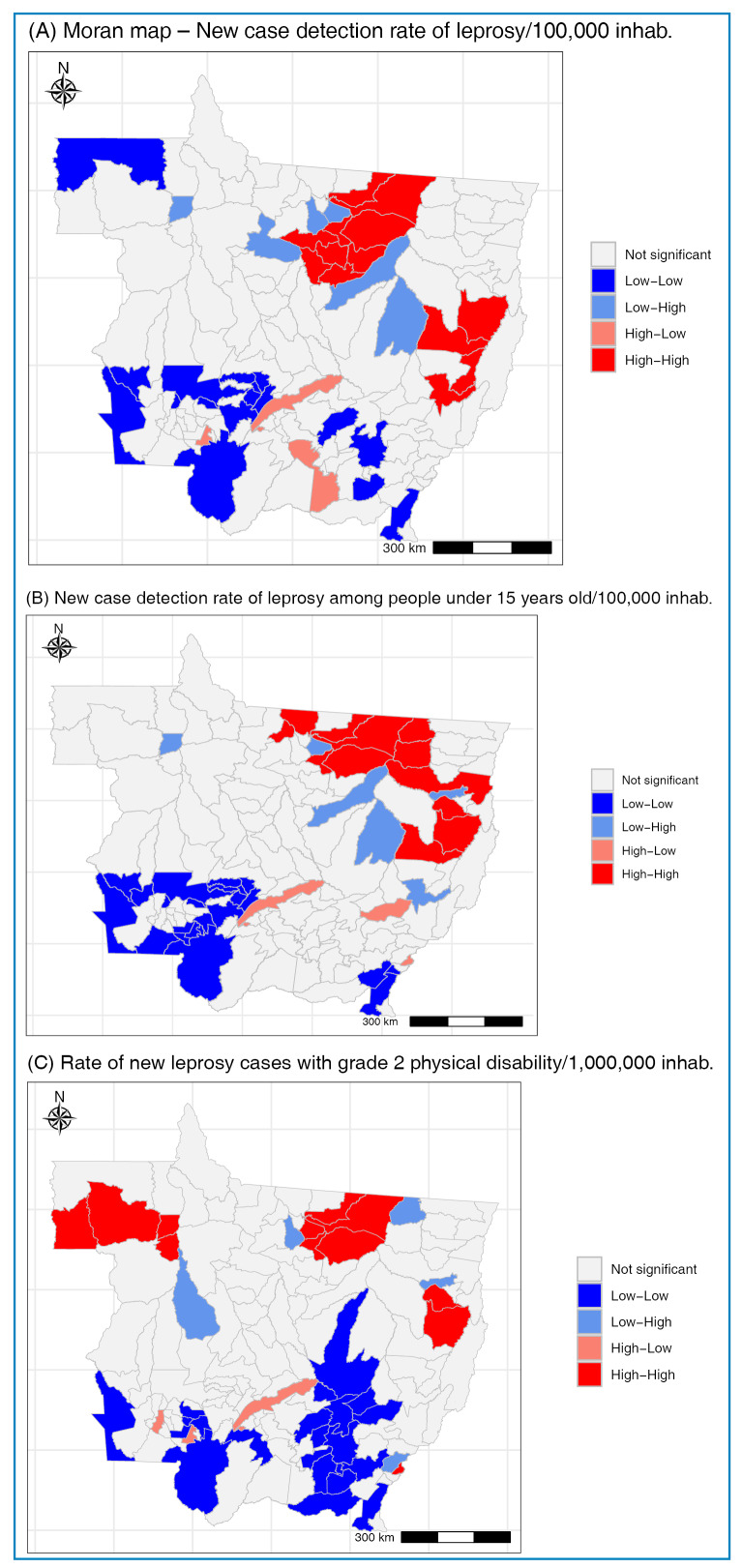
Spatial distribution of clusters of significant municipalities according to the selected epidemiological indicators of leprosy in Mato Grosso from 2008 to 2022^a^.

^a^Clusters estimated using the local indicators of spatial association cluster map statistic.

A spatial autocorrelation test was conducted when the leprosy epidemiological indicators became available. The global Moran’s index confirmed the spatial dependence between municipalities for all three indicators: NCDR (global Moran’s = 0.348; p = 0.001), NCD15 (global Moran’s = 0.269; p = 0.001), and G2DR (global Moran's = 0.275; p = 0.001) (Figure 2). This finding indicates that the analyzed dataset demonstrated the formation of clusters. The “high-high” clusters (high risk of leprosy transmission) for the NCDR and NCD15 rates included 13 and 12 municipalities, respectively, located in the northern and eastern macroregions of MT. For G2DR, the “high-high” clusters comprised 12 municipalities located in the northwestern, northern, and eastern macroregions of the state.

## DISCUSSION

This study analyzed the spatial and temporal distributions of three leprosy surveillance indicators from 2008 to 2022 in the MT using robust spatial analysis and temporal trends. The primary results showed stability in the G2DR and a recent reduction (from 2018 to 2019) in the NCDR and NCD15 rates. However, high rates of these indicators persisted in the last few years of the analyzed series. Cluster analysis revealed significant internal inequalities in the distribution of these three indicators, particularly in the northern, northwestern, and eastern regions of the state. Notably, the municipalities of Peixoto de Azevedo, Sinop, Juína, Peixoto de Azevedo, and Ponte Branca were included in the significant clusters for these analyzed indicators.

Geoprocessing techniques, including the cluster grouping method used in this study, play an important role in exploratory data analysis and pattern recognition. These techniques can assist in planning disease surveillance and control actions^22^ ^-^ ^25^. In recent years, these techniques have been widely applied to describe the distribution characteristics and transmission patterns of leprosy in Brazil. However, only a few studies have used data from the MT, one of the most endemic areas for leprosy in the country^26^ ^-^ ^28^. The results demonstrated the usefulness of these tools in examining leprosy. The cluster analysis techniques employed in this study enabled the identification of areas with heightened epidemiological risk for leprosy in the MT, thereby allowing for the prioritization of interventions and further studies in these regions. Additionally, these techniques have been used to explore the spatial dynamics of leprosy distribution, monitor epidemiological indicators over time, and identify risk factors and clusters of high endemicity.

This study revealed that the detection rates of new leprosy cases in the general population and children aged <15 years (in 2022 in the MT) remain above the minimum parameters recommended by the Ministry of Health^14^. The high incidence of new leprosy cases in children aged <15 years suggests an ongoing transmission, highlighting the need for prevention strategies such as post-exposure prophylaxis with a single dose of rifampicin^4^ ^,^ ^29^. Additionally, active case detection and contact tracing are established strategies that contribute to the early detection of leprosy and the reduction of its transmission^4^ ^,^ ^6^. These results highlight the need for alerts to guide emergency actions in the state and facilitate more detailed investigations into their causes.

The study revealed a stable rate of new leprosy cases with grade 2 disability at the time of diagnosis, despite simultaneous decreases in the NCDR and TD indicators^15^. These results, observed in the context of the recent pandemic, may reflect operational failures in identifying new patients with leprosy, including those aged <15 years. This situation may explain the lack of a significant decrease in the proportion of patients exhibiting advanced disability at the time of diagnosis. This underscores the need to enhance the coverage and quality of epidemiological surveillance in MT, particularly in the detection of new cases and implementation of adequate and timely diagnosis and treatment^30^ ^,^ ^31^. According to Rodrigues (2020)^9^ and Martoreli Júnior (2021)^32^ and their collaborators, the effectiveness of these actions is hindered by barriers the lack of investment, workforce limitations, poor organizational management, and the lack of knowledge about the disease. Historically, leprosy has been linked to social stigma, which compromises the mental health of individuals affected by the disease, leading to psychological distress, social isolation, and diminished ability to work^31^ ^,^ ^33^. This psychological impact on individuals with leprosy can lead to reluctance to seek appropriate treatment, thereby contributing to the advancement of the disease and leading to peripheral neuropathy, potential disability, and stigma^33^ ^,^ ^34^.

Using the global Moran’s index, we identified spatial dependence among the municipalities for the three indicators analyzed. This analysis revealed that the leprosy burden was unevenly distributed, forming significant clusters of high transmission and advanced disease at the time of diagnosis. Other studies have corroborated that leprosy has a heterogeneous spatial pattern, with specific areas having a high epidemiological risk^26^ ^,^ ^27^ ^,^ ^35^.

This study identified the persistence of certain municipalities and their surrounding areas within high-risk clusters for the indicators analyzed, particularly Peixoto de Azevedo, Sinop, Juína, Ribeirãozinho, and Ponte Branca. These municipalities highlight the North, East, and Northwest regions of MT. These areas, with the most critical indicators noted in this study, have already been recognized in the literature as important endemic regions in MT^8^ ^,^ ^28^. A study conducted in the municipality of Alta Floresta (MT), located in the northern macroregion of MT, between 2016 and 2018, revealed a predominance of multibacillary cases, a high rate of late diagnoses, and ongoing disease transmission^28^. The authors underscored the importance of systematic contact tracing, screening, and the administration of a single dose of rifampicin to the contacts of patients with leprosy to control the disease. Another study examined the factors associated with the average detection rate of new leprosy cases in Brazilian municipalities and revealed that municipalities with the worst socioeconomic indicators and the highest levels of social inequality had a higher incidence of the disease^36^. Disordered growth, particularly linked to mining and agricultural settlements in Amazonian municipalities, has resulted in the emergence of poverty pockets concentrated in territories often overlooked in aggregate analyses. The northern, northwestern, and eastern regions of MT are characterized by low economic development and high social vulnerability^37^.

The municipalities classified as “low-low” clusters should be analyzed with caution as their average rates exceeded the parameters recommended by the Ministry of Health and, in many cases, showed an upward trend between the periods analysed^14^. Furthermore, the results of these municipalities can be partially attributed to the under-reporting of cases and require further evaluation.

This study has several limitations that must be considered. One limitation is the use of secondary data, which may contain inconsistencies, standardization failures, and quality issues, and the underreporting or absence of relevant information. Additionally, the impact of the COVID-19 pandemic on notifications and care in 2020-2021. To minimize this influence, we excluded these atypical years from some of the analyses. Some findings may be partially attributed to barriers to accessing health services, which were exacerbated during the pandemic period, leading to the underdetection of cases. In non-pandemic years, the underreporting and underdetection of leprosy cases have been documented in microregions where the disease is diagnosed less frequently, such as in the South and Southeast Regions. This pattern may have shifted during the pandemic^38^. Another limitation is the use of crude rates in *cluster* analyses, which can lead to instability in estimating the epidemiological risk, particularly in municipalities with small populations. Finally, using the municipality as the unit of analysis can obscure internal, intramunicipal inequalities, especially in large municipalities with diverse social determinants. When possible, the use of even smaller geographical units of analysis can help identify high-risk areas within municipalities that may appear to be at low or medium risk.

We recommend intensifying effective measures to enhance access to early diagnosis of the disease. This includes training medical professionals in the proper diagnosis and management of these patients, implementing leprosy prevention and control actions in areas with high epidemiological risk based on the identified clusters, and providing continued support for epidemiological and evaluative research to better understand the factors associated with disease persistence and the effectiveness of the implemented interventions.

In conclusion, the results of this study contribute to the identification of diverse epidemiological scenarios for leprosy in MT. These insights not only facilitate a more efficient allocation of resources but also pave the way for more targeted epidemiological surveillance and the formulation of public policies aimed at disease control. The use of these analytical tools to monitor leprosy incidence is essential for enhancing the effectiveness of health management strategies within the region.
